# Contrast-enhanced mammography predicts pathological response after neoadjuvant chemotherapy in locally advanced breast cancer

**DOI:** 10.3332/ecancer.2022.1396

**Published:** 2022-05-23

**Authors:** Daniel Canteros, Benjamin Walbaum, Miguel Córdova-Delgado, Andrés Torrealba, Constanza Reyes, María Elena Navarro, Dravna Razmilic, Mauricio Camus, Francisco Dominguez, Orieta Navarrete, Mauricio P Pinto, Gonzalo Pizarro, Francisco Acevedo, César Sánchez

**Affiliations:** 1 Department of Hematology-Oncology, Faculty of Medicine, Pontificia Universidad Católica de Chile, Santiago 8330024, Chile; 2 Department of Surgical Oncology, Faculty of Medicine, Pontificia Universidad Católica de Chile, Santiago 8330024, Chile; 3 Department of Radiology, Faculty of Medicine, Pontificia Universidad Católica de Chile, Santiago 8330024, Chile; 4 Department of Pathology, Faculty of Medicine, Pontificia Universidad Católica de Chile, Santiago 8330024, Chile

**Keywords:** contrast-enhanced mammography, neoadjuvant chemotherapy, pathological complete response, sensitivity and specificity

## Abstract

**Introduction:**

Recently, contrast-enhanced mammography (CEM) has emerged as a reliable alternative to breast magnetic resonance imaging (MRI) for the assessment of pathological response in breast cancer patients. Our study sought to determine the diagnostic accuracy of CEM to predict pathological complete response (pCR) in patients who received neoadjuvant chemotherapy (NACT).

**Methods:**

We retrieved the medical records of patients who underwent NACT at our institution. Using post-surgery pCR, morphological evidence and CEM enhancement tumours were classified as follows: 1) radiologic complete response (rCR); 2) functional radiological complete response (frCR); and 3) non-complete response. Initially, we used multivariate analyses adjusted by clinical variables and frCR or rCR to determine which variables affected pathological response. Then, CEM diagnostic accuracy to discriminate pCR was assessed using receiver operating characteristic curves in univariate and multivariate models including either frCR or rCR.

**Results:**

A total of 48 patients were included in our study. Most patients (68.7%) were hormone receptor (HR)+ and 41.6% (20) of the patients achieved pCR. Using univariate logistic regression analyses we found that HR status, HER2 status, rCR and frCR had a significant impact on CEM diagnostic accuracy. Exploratory analyses found that CEM sensitivity was higher for HR− tumours. Multivariate logistic regression analyses found 60% sensitivity, 92.9% specificity and 79.2% accuracy in a model that included clinical variables and rCR.

**Conclusion:**

CEM is a reliable alternative to high-cost, time-consuming breast MRI that predicts pCR in patients undergoing NACT; CEM diagnostic accuracy was higher among patients who harboured HR− tumours.

## Introduction

Recent global estimates indicate that female breast cancer (BC) is the most commonly diagnosed malignancy, surpassing lung cancer with >2.3 million new cases in 2020 [1]. The majority of newly diagnosed BC cases (>90%) correspond to non-metastatic disease (stages I/II/III). The standard of care in these patients involves surgery and often some form of chemotherapy (CT) [2]. In recent years, preoperative neoadjuvant CT (NACT) has become the mainstay of treatment for locally advanced disease. This strategy not only reduces tumour size and micrometastatic foci but also offers a window of opportunity to assess chemosensitivity in patients, providing prognostic information [3–5]. Additionally, the presence of residual disease can guide subsequent treatment decisions [6, 7]. 

For decades, the response to NACT has been traditionally evaluated by conventional imaging techniques such as mammography and ultrasound (US). Unfortunately, these methods usually fail to predict the final outcome in terms of pathological response of patients. More recently, breast magnetic resonance imaging (MRI) has been proven superior to these techniques and has consequently become the preferred choice for the assessment of disease burden and for an accurate quantification of residual tumour following NACT [8]. While both US and mammography are restricted to morphological evaluations, breast contrast-enhanced MRI (CE-MRI) also integrates functional tissue properties and pathophysiological characteristics of the tumour including angiogenesis and leaky microvasculature [9]. However, given its elevated costs and limited availability, patient access to CE-MRI is somewhat restricted [10]. Alternatively, contrast-enhanced mammography (CEM) is an emerging modality that combines conventional mammography with iodinated contrast, improving detection and delivering morphological and functional characteristics of the tumour. Importantly, studies also demonstrate that CEM offers a faster procedure that implies less discomfort and anxiety [3, 11]. Despite this, the evidence on preoperative CEM is scarce.

Herein, we deliver a retrospective analysis of BC patients who underwent NACT, followed by preoperative CEM, describing sensitivity and specificity post-NACT for pathological complete response (pCR) according to clinicopathological BC subsets.

## Materials and methods

### Study design and ethics approval

This was a retrospective study conducted at the Cancer Centres of Pontificia Universidad Católica de Chile. All procedures strictly adhered to ethical standards contained in the Declaration of Helsinki (updated in 2013). These were also reviewed and approved by the Human Research Ethics Committee at our institution (School of Medicine, Pontificia Universidad Católica de Chile; approval resolution number: 200303006).

### Patients and clinical data

Patients’ clinical characteristics, images and post-surgery pathological data were retrieved from medical records of patients diagnosed over the period 2015–2020 at the Pontificia Universidad Católica de Chile Cancer Centre in Santiago, Chile. Hormone receptor (HR) status was inferred from the pathology reports. Therefore, HR+ tumours were defined according to the American Society of Clinical Oncology (ASCO) – College of American Pathologists (CAP) criteria as those tumours that displayed >1% of positivity on nuclear staining for oestrogen or progesterone receptors in tumour cells, and ERBB2 positive (hereafter called HER2) as those presenting immunoscore +++ or ERBB2 amplified by fluorescent *in situ* hybridisation [12]. Complete image response was defined as the absence of tumour contrast enhancement and morphological tumoural evidence.

### Imaging technique

Patients with an allergy to iodine were premedicated according to the institution’s protocol. In patients with kidney failure, >60 years, hypertensive and diabetic, plasma creatinine was previously requested (upper limit: 1.5 mg/dL).

CEM were performed using a SenoBright CESM device (GE Medical Systems, Buc, France). Iodinated contrast medium was i.v. administered at a dose of 1.5 ml/kg, using an injector at a speed of 3 mL/second prior to the acquisition of images (Mallinckrodt Optiray 320, Ioversol 320 mg at 68%) (Liebel-Flarsheim Company LLC, Raleigh, NC). Two minutes after injection, the patient was positioned, obtaining images of low (26 and 31 kVp) and high energy (45–49 kVp) successively in conventional projections (caudal skull and mediolateral oblique). The entire procedure lasted 10 minutes.

### Image processing

A reconstruction algorithm was applied to acquired images to subtract the parenchyma that do not enhance, obtaining a processed image with iodine enhancement.

### Pathological complete response (pCR)

All patients in this study underwent either conservative surgery or total mastectomy. Surgical specimens were evaluated by an expert pathologist following the ASCO CAP guidelines to evaluate residual disease; pCR was considered ypT0/TisN0M0 [13].

### Imaging interpretation

Images were reinterpreted on a high-resolution monitor (5-MP) by BC expert radiologists (>5 years of experience with CEM), assisted by previous images to compare blindly to histological results.

Image analyses were carried out considering the following parameters:

1- Any mammographic alteration in the low energy images (residual tumour, nodule, asymmetry or parenchymal distortion in the tumour bed).

2- Enhancement with the use of intravenous contrast in the recombined images, considering any enhancement as pathological.

Findings were categorised as the presence or absence, and the response to NACT was subsequently classified into three categories:

1. Radiologic complete response (rCR): no mammographic alterations or in the low energy and recombined images.
2. Functional radiological complete response (frCR): no contrast uptake, independent of morphological alterations.
3. No complete imaging response: any positive finding according to the parameters evaluated (includes partial response, progression and no response).

Regarding the quantification of residual disease, although it was calculated by measuring the maximum axis length (in mm), it was eventually categorised as a dichotomic variable, meaning presence or absence of residual disease. On the other hand, given that ypT0/Tis was considered a pCR, the presence/absence of microcalcifications was not included when we assessed the response to neoadjuvant treatments. However, they were taken into consideration for planification and extent of surgery and were obviously resected.

### Statistical analysis

First, baseline patient characteristics were analysed using descriptive statistics. Chi-square or analysis of variance tests were used to compare categorical or continuous variables, respectively. The association between CEM enhancement and clinicopathological variables with pCR was analysed using univariate logistic regression models, reporting odds ratio (OR) values with 95% confidence interval (95% CI). Multivariate logistic regression models were built incorporating significantly associated variables obtained in the univariate analysis. Finally, we evaluated the capacity of uni/multivariate logistic regression models in predicting pCR. The predictive capacity was evaluated by the area under curve (AUC) from the receiver operating characteristic (ROC) curve, specificity, sensitivity, accuracy, positive predictive value and negative predictive value metrics. CIs of ROC curves were computed using a 1,000-bootstrap resampling method. A *p*-value < 0.05 was considered indicative of a significant difference. We used R version 4.1.0 including ‘arsenal’, ‘knitr’, ‘ROCit’, ‘tidyverse’ and ‘dplyr’ packages for our analyses.

## Results

**Figure 1.**
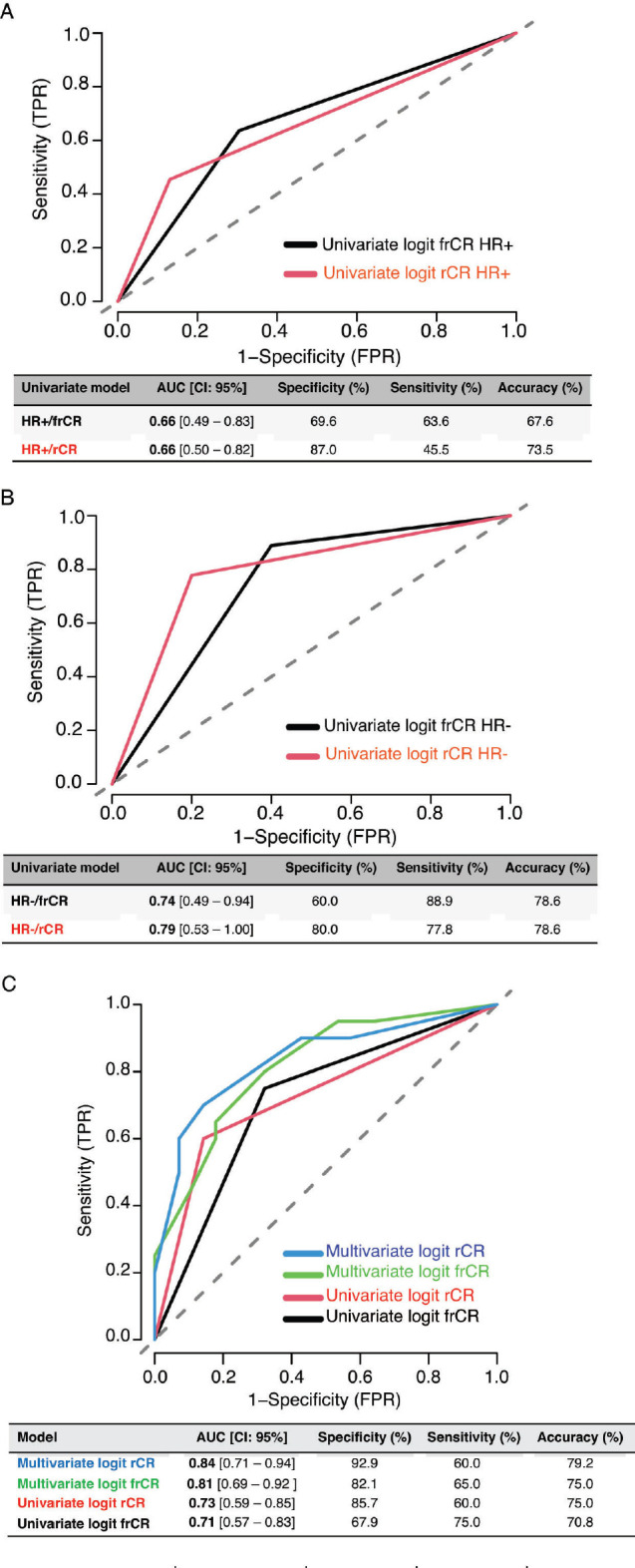
Diagnostic accuracy of CEM by HR status and ROC curves in univariate and multivariate models. (a) Diagnostic accuracy in HR+ tumours. (b) Diagnostic accuracy in HR− tumours. (c) ROC curves. Abbreviations: TPR: true positive rate, FPR: false positive rate, HR: hormone receptor, Logit: logistic regression, AUC: area under the curve, frCR: functional radiological complete response and rCR: radiologic complete response.

**Figure 2.**
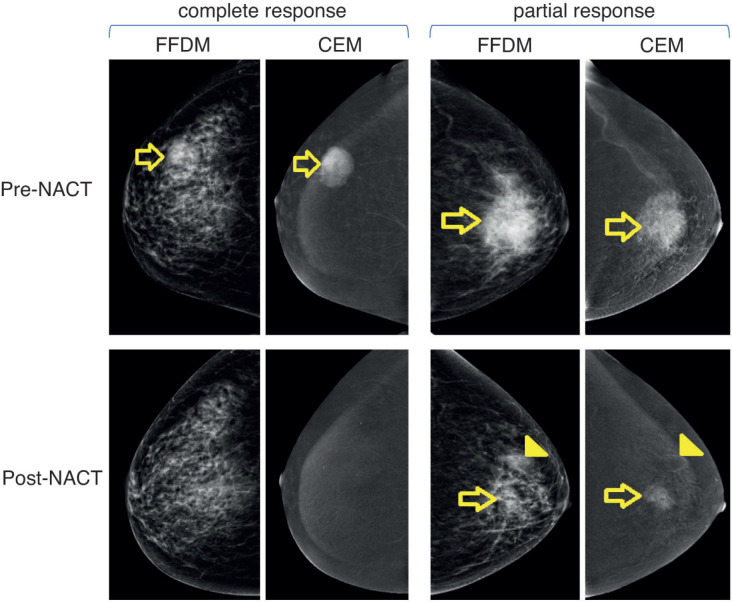
Examples of patients with complete or partial response to NACT. Left panels show images of a 51-year-old female patient with right breast invasive ductal carcinoma with a complete response to NACT. Upper and lower panels show pre-NACT and post-NACT images, respectively. Right panels show images of a 60-year-old female patient with left breast invasive ductal carcinoma with residual disease (partial response) after NACT. Abbreviations: FFDM: full-field digital mammography, CEM: contrast-enhanced mammography; and NACT: neoadjuvant chemotherapy.

A total of 48 patients who underwent NACT between 2015 and 2020 were included into our study. The main characteristics are summarised in Table 1. In brief, median age at diagnosis was 47 years old (range: 29–74.4 years). As expected, most cases were HR+ (70.8%) and the most frequently used NACT regimen was sequential anthracyclines, followed by taxanes (85.1%). A total of 20 patients (41.6%) achieved pCR. First, we sought to determine which factors were associated with achieving pCR and performed a univariate analysis, including age at diagnosis, stage at diagnosis, HR status, HER2 status, rCR and frCR. Table 2 shows that HR/HER2 status (hereafter called ‘clinical variables’), frCR and rCR had a significant impact on patients’ pCR. Next, we further confirmed the effect of these variables using multivariate analyses adjusted by clinical variables and frCR or rCR accordingly (Table 3). Then, as an exploratory analysis, we evaluated the CEM diagnostic accuracy to discriminate pCR using either frCR or rCR. Therefore, we built ROC curves and obtained predictive scores. First, we compared frCR versus rCR in HR+ or HR− tumours (Figure 1A and B, respectively). Although we did not find significant differences between frCR and rCR, AUC values were higher on HR+. Interestingly, specificity values in HR+ tumours were also higher; however, sensitivity values were lower compared to HR− tumours, particularly for rCR. Next, we analysed the entire cohort and did not find differences between AUC values for frCR and rCR: 0.71 (95% CI: 0.57–0.83) and 0.73 (95% CI: 0.59–0.85), respectively. We further confirmed that rCR has less sensitivity compared to frCR (60.0% versus 75.0%), but higher specificity (85.7% versus 67.9%). Overall, accuracy was slightly better with rCR versus frCR (75.0% versus 70.8%). As expected, multivariate models (Figure 1C) that included HR and HER2 status, and rCR or frCR displayed higher AUC values: 0.84 for rCR (95% CI: 0.71–0.94) and 0.81 for frCR (95% CI: 0.69–0.92). Notably, the rCR model displayed a high specificity (92.9%) and accuracy (79.2%). However, sensitivity remained at 60%, similar to the univariate model. Finally, Figure 2 shows examples of CEMs in patients with complete (left panels) or partial (right panels) responses.

## Discussion

In line with recent reports, our study confirms CEM is a reliable alternative to high-cost, time-consuming breast MRI for an accurate assessment of NACT response [14, 15]. Our data also demonstrate that CEM can predict post-NACT pathological response across BC subsets with high specificity (92.9%) and moderate sensitivity (60%), Sensitivity increases in our enhancement-based response (frCR) analyses (65%), maintaining accuracy (79.2% and 75% in frCR and rCR, respectively). Our findings also indicate that CEM sensitivity was higher in HR–BCs (88.9% versus 63.6% in HR+ BC). Previous prospective studies have reported specificity levels between 83% and 88% and sensitivity values ranging from 76% to 100% [16–18]. However, in all cases, these reports included a lower number of cases compared to our study. Differences can also be attributed to the distribution of BC subtypes in their samples, the criteria to define pCR and/or technical differences, such as the definition of imagenological response. Interestingly, a retrospective study by Patel *et al* [11] that included a total of 65 patients compared CEM versus MRI and found similar levels of specificity and sensitivity (67% versus 69% and 95% versus 95%, respectively).

As previously described for breast MRI, CEM performance was also affected by tumour biology [19]. Indeed, CEM displayed higher sensitivity among HR− tumours, especially when using enhancement response criteria (rCR). This is probably explained by higher proliferation rates and increased angiogenesis in triple negative and HER2+ tumours compared to luminal-type tumours [9, 19].

Surprisingly, median age at diagnosis in our cohort was 47 years and 68% of the patients were <50 years old. On the contrary, local reports indicate a median age at diagnosis of 56 years among Chilean patients [20]. We speculate this is due to an over representation of HR− tumours in our cohort which are generally diagnosed at a younger age. In this regard, it is reasonable to speculate that CEM accuracy will be higher in older populations, since these tumours usually display lower breast densities [21]. Future studies should evaluate this possibility.

Another important consideration is related to the use of taxanes in our cohort. These are antiangiogenic compounds that suppress microvascular permeability and thereby decrease blood flow. Therefore, they might cause a reduction in enhancement, decreasing CEM’s sensitivity, regardless of patient’s oncologic response [9]. All patients in our study received taxanes either in combination or as monotherapy. Therefore, taxane was the last cytotoxic drug prior to image control. Future studies should explore the impact of treatments that start with weekly paclitaxel, followed by anthracyclines plus cyclophosphamide on CEM’s specificity and sensitivity.

Our work has several limitations that are mainly derived from its retrospective nature. First, we lack information regarding selection criteria for either CEM or not MRI by the surgeon and whether it was determined according to clinical risks. Second, we dichotomised HR expression (+ or −); however, CEM accuracy probably varies continuously according to HR expression. In this regard, studies suggest that tumours that express low levels of oestrogen and progesterone receptors behave like HR-negative BCs [19]. Despite this, basic patient characteristics, including stage and subtype distribution, were in line with previous reports [18], even though these studies used a different definition of pCR. Although our study independently evaluated functional response (intravenous contrast (IVC) on recombined images), the use of CEM in the clinical practice should always be interpreted along with low energy images that reveal morphological alterations and the amount of contrast uptake on recombined images. Finally, technical failures or adverse reactions were not determined in our study.

## Conclusion

In summary, despite the use of different imagenological criteria, dissimilar definitions of pCR and BC subtype distributions, our findings confirm CEM’s diagnostic and predictive performance reported by others. Our data also encourages the use of CEM as a substitute for high-cost/time-consuming breast MRI, especially in patients who harbour HR− tumours. Evidently, future clinical trials should further validate and confirm these findings and elucidate the precise role of BC subtype in this setting.

## Conflicts of interest

None.

## Funding

This research did not receive any specific grant from funding agencies in the public, commercial or not-for-profit sectors.

## Figures and Tables

**Table 1.** Clinicopathological characteristics of patients.

		Non-pCR (*n* = 28)	pCR (*n* = 20)	Total (*n* = 48)	
Variables		Median (range) or n (%)	Median (range) or n (%)	Median (range) or n (%)	*p*-value
Age		47.4 (29.6–68.2)	46.2 (29.0–74.4)	47.0 (29.0–74.4)	0.59
Age	< 50 years	17 (60.7)	11 (55.0)	28 (58.3)	0.69
	≥ 50 years	11 (39.3)	9 (45.0)	20 (41.7)	
Stage at diagnosis	II	15 (53.6)	14 (70.0)	29 (60.4)	0.25
	III	13 (46.4)	6 (30.0)	19 (39.6)	
HR status	(−)	5 (17.9)	9 (45.0)	14 (29.2)	0.04*
	(+)	23 (82.1)	11 (55.0)	34 (70.8)	
HER2 status	(−)	19 (67.9)	7 (35.0)	26 (54.2)	0.02*
	(+)	9 (32.1)	13 (65.0)	22 (45.8)	
BC subset	HR+/HER2-	13 (46.4)	4 (20.0)	17 (35.4)	0.05*
	HR+/HER2+	9 (32.1)	7 (35.0)	16 (33.3)	
	HR-/HER2+	1 (3.6)	6 (30.0)	7 (14.6)	
	HR-/HER2-	5 (17.9)	3 (15.0)	8 (16.7)	
Type of CT	AT	25 (89.3)	15 (78.9)	40 (85.1)	0.11
	ATC	1 (3.6)	1 (5.3)	2 (4.3)	
	T	2 (7.1)	0	2 (4.3)	
	TC	0	3 (15.8)	3 (6.4)	
frCR	No	9 (32.1)￼	15 (75.0)	24 (50)	0.003*
	Yes	19 (67.9)	5 (25.0)	24 (50)	
rCR	No	4 (14.3)	12 (60.0)	16 (33.3)	< 0.001*
	Yes	24 (85.7)	8 (40.0)	32 (66.7)	

HR: Hormone receptor, HER2; Human epidermal growth factor receptor 2; BC: Breast cancer; AT: Anthracycline/taxane, ATC: Anthracycline/taxane/cyclophosphamide, T: Taxane, TC: Taxane/cyclophosphamide, frCR: Functional radiological complete response and rCR; Radiologic complete response.

* indicates *p* < 0.05

**Table 2.** Univariate logistic regression for pCR using clinical and imaging variables.

		Univariate logistic regression	
Variables		OR (CI: 95%)	*p*-value
Age	< 50 years	Ref.	0.69
	≥ 50 years	1.26 (0.39–4.08)	
Stage at diagnosis	II	Ref.	0.25
	III	0.49 (0.14–1.62)	
HR status	(−)	Ref.	0.04*
	(+)	0.27 (0.07–0.95)	
HER2 status	(−)	Ref.	0.02*
	(+)	3.92 (1.20–13.89)	
frCR	No	Ref.	0.003*
	Yes	0.16 (0.04–0.54)	
rCR	No	Ref.	< 0.001*
	Yes	0.11 (0.02–0.41)	

HR: Hormone receptor, HER2: Human epidermal growth factor receptor 2, OR: Odds ratio, Ref: Reference, pCR: Pathological complete response: frCR: Functional radiological complete response and rCR: Radiologic complete response.

* indicates *p* < 0.05

**Table 3.** Multivariate logistic regression for pCR adjusted by clinical variables and frCR or rCR.

		Multivariate (clinical + frCR)		Multivariate (clinical + rCR)	
Variables		OR (CI: 95%)	*p*-value	OR (CI: 95%)	*p*-value
HR status	(−)	Ref.	0.11	Ref.	0.16
	(+)	0.28 (0.05–1.30)		0.31 (0.05–1.60)	
HER2 status	(−)	Ref.	0.02*	Ref.	0.02*
	(+)	5.37 (1.34–26.62)		6.26 (1.47–35.56)	
frCR or rCR	No	Ref.	0.02*	Ref.	0.007*
	Yes	0.18 (0.04–0.70)		0.11 (0.02–0.50)	

HR: Hormone receptor, HER2: Human epidermal growth factor receptor 2, OR: Odds ratio, Ref: Reference, pCR: Pathological complete response, frCR: Functional radiological complete response and rCR: Radiologic complete response.

* indicates *p* < 0.05

## References

[1] Sung H, Ferlay J, Siegel RL (2021). Global Cancer Statistics 2020: GLOBOCAN estimates of incidence and mortality worldwide for 36 cancers in 185 countries. CA Cancer J Clin.

[2] Castillo CSM, Cabrera MEC, Derio PL (2017). Resultados del tratamiento del cáncer de mama, programa nacional de cáncer del adulto. Rev Med Chil.

[3] Fowler AM, Mankoff DA, Joe BN (2017). Imaging neoadjuvant therapy response in breast cancer. Radiology.

[4] King TA, Morrow M (2015). Surgical issues in patients with breast cancer receiving neoadjuvant chemotherapy. Nat Rev Clin Oncol.

[5] Rastogi P, Anderson SJ, Bear HD (2008). Preoperative chemotherapy: updates of national surgical adjuvant breast and bowel project protocols B-18 and B-27. J Clin Oncol.

[6] von Minckwitz G, Huang CS, Mano MS (2019). Trastuzumab emtansine for residual invasive HER2-positive breast cancer. N Engl J Med.

[7] Masuda N, Lee SJ, Ohtani S (2017). Adjuvant capecitabine for breast cancer after preoperative chemotherapy. N Engl J Med.

[8] Berg WA, Gutierrez L, NessAiver MS (2004). Diagnostic accuracy of mammography, clinical examination, US, and MR imaging in preoperative assessment of breast cancer. Radiology.

[9] Lee-Felker SA, Tekchandani L, Thomas M (2017). Newly diagnosed breast cancer: comparison of contrast-enhanced spectral mammography and breast MR imaging in the evaluation of extent of disease. Radiology.

[10] Patel BK, Gray RJ, Pockaj BA (2017). Potential cost savings of contrast-enhanced digital mammography. Am J Roentgenol.

[11] Patel BK, Hilal T, Covington M (2018). Contrast-enhanced spectral mammography is comparable to MRI in the assessment of residual breast cancer following neoadjuvant systemic therapy. Ann Surg Oncol.

[12] Wolff AC, Hammond MEH, Allison KH (2018). Human epidermal growth factor receptor 2 testing in breast cancer: American society of clinical oncology/ college of American pathologists clinical practice guideline focused update. J Clin Oncol.

[13] FDA (2012). Pathologic Complete Response in Neoadjuvant Treatment of High-Risk Early-Stage Breast Cancer: Use of an Endpoint to Support Accelerated Approval.

[14] Sensakovic WF, Carnahan MB, Czaplicki CD (2021). Contrast-enhanced mammography: how does it work?. Radiographics.

[15] Jochelson MS, Lobbes MBI (2021). Contrast-enhanced mammography: state of the art. Radiology.

[16] Barra FR, Sobrinho AB, Barra RR (2018). Contrast-enhanced mammography (CEM) for detecting residual disease after neoadjuvant chemotherapy: a comparison with breast magnetic resonance imaging (MRI). Biomed Res Int.

[17] Iotti V, Ravaioli S, Vacondio R (2017). Contrast-enhanced spectral mammography in neoadjuvant chemotherapy monitoring: a comparison with breast magnetic resonance imaging. Breast Cancer Res.

[18] ElSaid NAES, Mahmoud HGM, Salama A (2017). Role of contrast enhanced spectral mammography in predicting pathological response of locally advanced breast cancer post neo-adjuvant chemotherapy. Egypt J Radiol Nucl Med.

[19] Zhang X, Wang D, Liu Z (2020). The diagnostic accuracy of magnetic resonance imaging in predicting pathologic complete response after neoadjuvant chemotherapy in patients with different molecular subtypes of breast cancer. Quant Imaging Med Surg.

[20] Walbaum B, Puschel K, Medina L (2021). Screen-detected breast cancer is associated with better prognosis and survival compared to self-detected/symptomatic cases in a Chilean cohort of female patients. Breast Cancer Res Treat.

[21] Mori M, Akashi-Tanaka S, Suzuki S (2017). Diagnostic accuracy of contrast-enhanced spectral mammography in comparison to conventional full-field digital mammography in a population of women with dense breasts. Breast Cancer.

